# Supplementary material for: The genome regulatory landscape of Atlantic salmon liver through smoltification
Source: PLoS One. 2024 Apr 22;19(4):e0302388. doi: 10.1371/journal.pone.0302388 (PMC11034671; doi:10.1371/journal.pone.0302388)
Supplement: Attachment — Submitted filename: revision_Harvey_Gillard_et_al.docx [file pone.0302388.s014.docx]

Harvey and Gillard et al. provide a nice experimental set up with multi-omic resouces – RNA-seq, ATAC-seq and methylation- to understand the changes at these three levels during salmon smoltification. Here I attach my comments with the aim to help in providing a more global vision on the processes taking place on salmon smoltification as well as to provide a better understanding of the changes during chromatin accessibility.

Major comments:

- The authors performed KEGG pathway analysis but I am no Gene Ontology enrichment. Would it add extra knowledge on the set of genes we are looking at each cluster?
- Data availability for ATAC-seq PREJEB65073 I could not find the fastq files (26/10/2023).
- The authors use Figure2 to later focus on lipid metabolism. However, I could not read any of the KEGG definitions. A better Figure 2 is required.
- In Figure 2 A we have per week 4 time columns. It would be good to mention in the figure legend they belong to biological replicates. Why in week 10 only two columns?
- Figure 2 A: “Relative liver” expression of genes… is that TPM per condition? It is ok, but for visualization purposes would not be better log2 TPM? TPM tend to have much higher range and log2 would help in the scaling…. (optional)
- When the authors compare gene expression changes between experimental group and freshwater group at week 25 they find 2,121 DEGS… Any information GO or KEGG analysis on those genes? Or at least with the “significantly?” overlapping in cluster 1 and cluster 2? Are they also overlapping other clusters but non-significantly? Any overall picture on GO / KEGG of the 281 or 259 genes before focusing on lipid metabolism? Does lipid metabolism keeps coming back?
- Why that major focus on lipid metabolism? Is there something else of interest?
- ATAC-seq: The authors find that PC1 splits weeks 1 and 19 from 10 and 25. Was this expected? Is there any information or are they having a batch effect or unwanted variation that could be checked using for example RUV-seq package from R and assess whether PC1 correlates with metadata information?
- I find Figure 7 very interesting but I do not fully understand the analysis. Are the authors providing the TFBS to gene association? Do they put a limit in the number of KB to closest gene? Could they describe in a more extensive manner the analysis? Thank you in advance.
- For the ATAC-seq analysis the authors have performed TOBIAS and motif enrichment. However, to give a sense of how much chromatin remodelling is taking place… would not be nice to perform a Differentially Accessiblity Analysis? That is to count the number of reads with the 201K peak set using for example featureCounts… and then treat the data as gene expression data… DESEQ2 or another methodology so we can get how many differentially regions are in each stage? Are DARS mostly enriched for proximal or distal regions?
- Also the authors can try to integrate then MA plots from RNA-seq and ATAC-seq to see if there is some link between open-chromatin and gene expression changes? For example, as in Mohammed et al. BMC genomics 2022 doi: 10.1186/s12864-022-08514-8 PMID: 35874611
- The authors comment of lipid metabolism genes and I think they have very good quality RNA-seq and ATAC-seq profiles… could the authors show some IGV tracks on some of lipid metabolism genes? To see if the ATAC-seq profile and RNA-seq goes down? It would be much more visual.
- Have the authors made available the salmon blacklisted file of repetitive regions?

Minor comments:

- Page 7 line 152 .. acsl3, and three acsl3 … should be acsl3 and four acsl4 ?
- Authors refer to figure 3B before figure 3A .
- Page 7 Line 156 … which reference table ? I think dgat1 and gat2 are not in the fig 3 B? which table was used to generate figure 3b?
- Page 8 Line 169/170… dgat1 and dgat2 …. Decreasee in fresheater smolts… where ? which figure or reference table?
- Page 8 Line 176 and 177 is the overlap significant? “Significantly overlap…” to check by hypergeometric test?
- Page 8: Line decreased expression fa1 and fas2 how much? Log2 FC and p-value?
- Page 8 Line 195 on D25-hydroxialse gene… again how much down-regualted log2 FC? Also for the 532 how many up-/down-regulated?
- Page 9 Line 214… is there any summary stats on the ATAC-seq data? Initial number of reads/ mapping rate, % duplicatated reads, % MT reads, FROT, FRIP ?
- Page 20; Typo 🡪 JASPER database to JASPAR database?
