# Supplementary material for: The genome regulatory landscape of Atlantic salmon liver through smoltification
Source: PLoS One. 2024 Apr 22;19(4):e0302388. doi: 10.1371/journal.pone.0302388 (PMC11034671; doi:10.1371/journal.pone.0302388)
Supplement: Attachment — Submitted filename: Comments to reviewers.docx [file pone.0302388.s015.docx]

Response to reviewers:

We thank the reviewers for their valuable feedback. Please find our point-by-point response to the reviewers’ comments below (response in blue).

Editor comments

1. The summary of fold change (up vs down), patterns of expression etc (see rev 1).

We added a table of expression fold change values for lipid genes specifically (Table 1) to add more details, along with specific fold change value when we mention other gene expression change.

2. Disucss the limits of the study, only one tissue - liver- why important, 2 replicates only for ATAC, quality of gene annotations, other issues?

We have added some additional justification to the use of liver and the focus on lipid metabolism to the introduction (lines 76-81). Regarding using only two ATAC-seq replicates, we do not believe that this has impacted our analysis due to the reproducibility of the ATAC data in the PCA (Fig 5A). Also, using two replicates for ATAC-seq is common practice and has been established as a standard by the ENCODE consortium (<https://www.encodeproject.org/data-standards/atac-seq/atac-encode4/>). For this reason, we do not view having 2 replicates as a limit of the study and do not think it is necessary to add a discussion on this.

3. Rev 1 raised concerns about the transition from global analyses to Lipid genes summary. Perhaps better highlight the functional diversity in this section, and better justify why focusing on lipids?

We have added some justification for the focus on lipid metabolism to the introduction (lines 76 to 81). We also identified and corrected an error when describing the enrichment trends in cluster 4 and 5 in figure 2 (lines 135-138). Additionally, we have modified the discussion on metabolic remodeling to include amino acid and carbohydrate pathways since we also observe gene expression in these pathways decrease in freshwater smolts before seawater transition (lines 372 to 374).

4. Rev 1 calls for “Better explanation of methods, namely integrative analysis motif discovery and RNA-seq”, this can be accommodated in part with supplemental texts.

We reworded the explanation of this analysis in the relevant method section (line 582 to 589).

5. Is there a technical reason for why timepoints 1 n 19 split from 10 n 25 on PC for the ATAC? Where all samples sequenced on same chip, or otherwise processed in parallel?

All ATAC-seq samples were sequenced together and processed in parallel. We do not anticipate a technical reason for this.

6. Recommend adding a designated conclusion section. That summarizes better the molecular details (insights) in the context of the physiology of smoltification and the photoperiod question. The abstract could also gain from such editing, because in current state it ends with deep molecular focus, without referencing back to the main questions posed.

We have added a dedicated conclusions section and modified the end of the abstract to tie the work together.

Reviewer 1

Reviewer #1: I believe the datasets are relevant and quite unique for in salmon species. However, I would recommend a major revision to :
a) be more accurate when they mention up-down regulation (add log2FC) to be more specific

We have added a table (table 1) which shows the log fold change of all the lipid metabolism genes mentioned in the results. Log2FC values for other genes mentioned in the results has been added to the text.

b) Perform a bit of extra-analysis in ATAC-seq before passing to motif discovery to have a global view of open-chromatin changes

We thank the reviewer for this suggestion as we believe it has greatly improved our analysis. To better link changes in chromatin accessibility to gene expression we have added three panels to figure 5 to highlight changes in chromatin accessibility during winter (weeks 1 to 10), during smoltification (weeks 10 to 19), and during seawater transfer (weeks 19 to 25) (Fig 5F). We then identified the nearest genes to these differentially accessible peaks and find that the direction of differential accessibility largely matches gene expression trends (Fig 5G and H). For example, peaks decreasing in accessibility during smoltification (week 10 to 19) were linked to genes belonging to cluster 7 which decreased in expression throughout the experiment and were enriched in lipid metabolism genes. We have added this information to the results section (lines 238 to 254) and discussion (lines 421 to 425)

c) Revise to provide a major angle (if there is margin to do so ) rather than focusing on lipid metabolism

Since we see smoltification associated downregulation of genes in amino acid and carbohydrate metabolism in addition to lipid metabolism, we have extended our interpretation to include these in preparatory metabolic remodeling (lines 372 to 374). However, we still emphasize lipid metabolism due to its relative importance in salmon farming. To support this we have added some additional justification for the focus on lipid metabolism to the introduction (lines 76-81)

d) improve the readership by better ligating the different sections of the manuscript. Also will help for non-salmon (smolt) experts

We have changed the subtitle of the second results section to better match the style of the other section subtitles.

e) make sure data is fully available for the community (ATAC-seq)

We have uploaded the ATAC-seq data to the European Nucleotide Archive under accession number PRJEB72206. Additionally these are now available through array express under accession numbers E-MTAB-11746 (RNA-seq) and E-MTAB-13743 (ATAC-seq)

f) Better explanation of methods, namely integrative analysis motif discovery and RNA-seq

We have modified the methods section to better explain the analysis (lines 582 to 589).

Reviewer 2

The study presents the results of primary scientific research.
This is a very good primary scientific research article. Not only does it present RNA-seq, but also ATAC-seq and bisulfide sequencing results, which are very novel not just in Atlantic salmon but in most non-model species. The code has all been published, making this paper a valuable contribution to the field

Results reported have not been published elsewhere.
I could not find any of these results published anywhere, aside from its corresponding pre-print
Experiments, statistics, and other analyses are performed to a high technical standard and are described in sufficient detail.

The methods are clear; both the laboratory and bioinformatic pipelines are well-defined. The issue is that all the methodologies are used very independently. It's a shame that more attempts were not made to integrate the datasets; however, I would not consider this reason enough to not publish this work

We have better integrated the data by adding three panels to figure 5. These show differentially accessible ATAC peaks and genes associated with these. We find that chromatin status is linked to changes in gene expression.

Conclusions are presented in an appropriate fashion and are supported by the data.
The conclusion is that photoperiod does not seem to affect liver metabolism. This is presented appropriately, and all three different datasets seem well-described and analyzed. Although this is a negative result, it appears to be a good resource and an effective use of state-of-the-art omics. This work can be followed up on in the future

The article is presented in an intelligible fashion and is written in standard English.
Very well written, but could do with some proofreading, especially in the introduction.

We have made several small grammatical corrections in the introduction.

The research meets all applicable standards for the ethics of experimentation and research integrity.
It does

The article adheres to appropriate reporting guidelines and community standards for data availability.
All data is publically available with accession numbers.

Comments

74-76 Although gill physiology has received most attention in the smoltification literature, other organs such as the liver also undergo large changes in function upon smoltification and seawater migration, with large implications for key metabolic traits. Please justify why the gill has received more research attention. This conclusion should be supported by references or data. Would be best to refer to it as being more studied that received more attention.

We have changed “received most attention” to “been most studied”. We also added “due to its role in osmoregulation” and reference to Nisembaum et al. 2021 to clarify why gill is more studied in relation to smoltification in salmon.

77 reared on different diets converges after smoltification [12, 13].
Not very clear what you mean by converges, can you explain it better?

We have edited this section to make this point more clear. It now reads as follows: “…reared on diets containing high or low long chain polyunstaturated fatty acids (LC-PUFA) have distinct lipid profiles during the parr stage, then during smoltification the lipid profiles converge”

81 Confounded does not make a lot of sense, maybe use another word?

To make this more clear and direct we removed “smolfication and seawater transfer were confounded (i.e…) ” and rather directly state that smolts in freshwater were not sampled.

94 consider changing a to the.

We have made this change.

103 of the trial, but some mortality (8x fish) in one tank due to improper oxygenation after seawater transfer.
Would hypoxia not affect the results? Did you use this fish?

We sampled 6 weeks after the hypoxia incident and assume there is no effect from this. Additionally, we have multiple tanks to account for such tank effects. We changed this to “immediately after seawater transfer” to emphasize that the mortality took place just after SW transfer and not closer to the time of sampling.

212-213 For each time point across the 25 week experiment group we generated two replicate samples of ATAC-seq data from the same livers sampled for RNA214 seq, at a depth of 55-72M reads.
Using only two samples is not statistically robust. Ideally, at least three replicates are recommended for ATAC-seq. Given the complexity of setting up this experiment, having more replicates may not be possible. However, having fewer replicates makes it difficult to infer statistically meaningful results with ATAC-seq across different time points. Integration of methods could have helped a bit with this.

While we agree that three ATAC-seq replicates may be more statistically robust, we do not believe that having two has impacted our results given the reproducibility of the ATAC data in the PCA plot. Additionally, at least two replicates has been established as the standard by the ENCODE consortium (<https://www.encodeproject.org/data-standards/atac-seq/atac-encode4/>). Since we are comparing ATAC and RNA signals, it is more important to have ATAC-seq and RNA-seq data coming from the same biological samples, which we have carried out for this analysis.

Figures 2, 6, and 7 have low resolution and need improvement to make them clearer.

We are unsure why the figures have low resolution. We have re-uploaded the figures as EPS files to attempt to resolve this issue.

Journal Requirements:

When submitting your revision, we need you to address these additional requirements.

1. Please ensure that your manuscript meets PLOS ONE's style requirements, including those for file naming. The PLOS ONE style templates can be found at 
<https://journals.plos.org/plosone/s/file?id=wjVg/PLOSOne_formatting_sample_main_body.pdf> and 
<https://journals.plos.org/plosone/s/file?id=ba62/PLOSOne_formatting_sample_title_authors_affiliations.pdf>

We have modified the title page to match the formatting PLOS one formatting style.

2. In your Data Availability statement, you have not specified where the minimal data set underlying the results described in your manuscript can be found. PLOS defines a study's minimal data set as the underlying data used to reach the conclusions drawn in the manuscript and any additional data required to replicate the reported study findings in their entirety. All PLOS journals require that the minimal data set be made fully available. For more information about our data policy, please see <http://journals.plos.org/plosone/s/data-availability>.

"Upon re-submitting your revised manuscript, please upload your study’s minimal underlying data set as either Supporting Information files or to a stable, public repository and include the relevant URLs, DOIs, or accession numbers within your revised cover letter. For a list of acceptable repositories, please see <http://journals.plos.org/plosone/s/data-availability#loc-recommended-repositories>. Any potentially identifying patient information must be fully anonymized.

Important: If there are ethical or legal restrictions to sharing your data publicly, please explain these restrictions in detail. Please see our guidelines for more information on what we consider unacceptable restrictions to publicly sharing data: <http://journals.plos.org/plosone/s/data-availability#loc-unacceptable-data-access-restrictions>. Note that it is not acceptable for the authors to be the sole named individuals responsible for ensuring data access.

We will update your Data Availability statement to reflect the information you provide in your cover letter.

All data has been uploaded to ENA and Array express. Accession numbers have all been added to the data availability statement in the manuscript.

3. We are unable to open your Supporting Information file [Fig S1-3 and Table S1-9]. Please kindly revise as necessary and re-upload.

We are unsure why the supporting information could not be opened. We were able to open all supporting files from the PLOS one submission PDF file. To attempt to address this we have re-uploaded all supporting files in the revised submission and changed supplementary figure files to EPS format.
