# Supplementary material for: The genome regulatory landscape of Atlantic salmon liver through smoltification
Source: PLoS One. 2024 Apr 22;19(4):e0302388. doi: 10.1371/journal.pone.0302388 (PMC11034671; doi:10.1371/journal.pone.0302388)
Supplement: Attachment — Submitted filename: Comments to reviewers.docx [file pone.0302388.s016.docx]

Response to reviewers:

We thank the editor for their valuable feedback. Please find our point-by-point response to the comments below (response in blue).

Editor comments

1. Aspects of the experimental design are hard to glean from first pass reading. This includes both the text and layout of Figure 1 (see below) and analyses for figure 2. For the latter forn instance, is this just the comparison over the 4 timepoints in the “natural smoltification“ treatment (that is excluding the experimental treatments)?

Figure 1 has been changed with a diagram to better describe the experimental timeline. We removed the use of experimental and control group terminology throughout and replaced with more explicit descriptions of the sample groups (see comment 4).

2. The results switch several times from past to present tense. Past tense is a more common style, but both can work. Do read over and check.

We have read through the results section and changed present to past tense where appropriate.

3. The language in discussion does on occasion turn very assertive, but the fact is that the data are all correlative. I suggest adding a genera caveat on that, the data are correlative and while coherent do not represent formal experimental proof of said influence. Perhaps either in beginning or end of discussion.

We have added a caveat at the end of the discussion extending to future work.

4. The wording in the methods, (particularly the ATAC) section, is quite challenging. I recommend careful proofreading for both content and style. Also related, was the ATAC data from a previously conducted study? (see wording in Line 581)

We have read through the entire methods section and clarified wording where appropriate. More specifically, wording around the different groups in the feeding trial (previously experimental and control groups) was changed to be more explicit. We now refer to the groups with different photoperiods as short and long photoperiod groups and the fish that stayed in freshwater as the freshwater control group. This change has been implemented throughout the paper.

The data referred to on line 581 was data generated in the earlier section, not from another study. We have removed “previously generated” to avoid this confusion.

Minor points and edits.
Line 96.
Reword. “test if the photoperiodic history is a major factor impacts” to “test if the photoperiodic history influences…” to

We have made this change.

Line 110 and Figure 1. Legend.
Add some details. How many fish where reared in each tank, treatment combniation? How many timepoint/treatment combinations were sampled? How many where sampled for each method (Same fish sampled for both RNAseq and ATAC seq?) Clarify that the fishes reprsented are a random sample of the fish in tank/treatment. Add statistics on the size data (either the represented fish or the tank/treatment as a whole).

We have added details to the text and to the figure caption. We have also reorganized the figure to provide more information.

Line 132.
Maybe specify that edgeR is used “using an ANOVA-like test.”,. comparing the 4 or 7? experimental groups?

We have reworded this line to make it clear that the ANOVA-like test is being performed on the fish experiencing short photoperiod and seawater transfer.

Line 134
Can drop “major”.

We have dropped the word “major”.

Line 139.
Can you invert the order “To associate well defined metabolic or signaling processes to the different gene expression trends,”
“To study the function of differentially expressed clusters we…”

We have implemented this suggestion.

Line 141.
Why not start with cluster 1? Or rename them so they appear in order in text. Cluster 6 is not talked about.

We have moved the sentence about cluster 1 to the beginning and added a sentence on cluster 6 stating that no pathways were significantly enriched.

Figure 2. Legend.
“Global gene expression changes across life-stage.” Title can be more descriptive. Can apply to any organisms really.
Legend indicates 6 clusters, figure has 7.
We has added salmon to the figure legend title and fixed the number of clusters in the text.

Line 164.
Did all the genes in this lipid metabolism pathway have significantly altered expression? Were some not DE?

We made additions to Table 1 and Figure 3 to indicate the lipid genes that were also tested to be differentially expressed across in the timepoints (Figure 2 results).

Line 200
Italic “de novo”

We have fixed this.

Line 210
Consider placing the fish earlier in the sentence, it is the organism in focus “expression suggests a reduced capacity to synthesize fatty acids in liver of fish after transition to sea,”

We have changed fish to salmon to be more specific and moved it earlier in the sentence.

Figure 3 legend.
Does expression of all these genes differ signifcantly with time?

We added annotation of which genes are significantly different across timepoints, as mentioned above.

Line 234.
Check wording “were concentrated were called to represent regions” – maybe “defined as”?

We have changed “called to represent” to “defined as”.

Line 254
Check wording “with the nearest gene and counted the distribution of previously identified”

We have reworded this slightly to make it clearer.

Line 249
Impressive improvement of the representation and cool results. P values from statistical tests are however missing for many of those. Can be incorporated into text or figure 5 legend.

P-value cutoff for differentially accessible peaks (Fig 5F) is mentioned in the figure legend (FDR < 0.05). Marked proportions in Fig 5G that are significantly greater by Fisher’s exact test p-value <0.05 by asterisks, with note in legend.

Line 279
Drop “therefore”

We made this deletion.

Figure 6 legend.
The “regulation” variable in the Figure is poorly explained. Add details to legend. “Green: … ColourX:… “

We have added text to the figure legend to explicitly state which colors correspond to which environmental variables.

Line 287
Add the caveat that you are inferring TF function based on data from other Eukaryotes / metazoa.

We have added “in mammals” to this sentence because the references we use are from studies in mammals.

Line 309
Rephrase “Next, we wanted to link TF binding patterns to the specific gene” perhaps.
“Next we tested for a link between TF binding patterns and gene…”

We have made this change.

Line 320.
Check wording “had nearby TFBS with NFIX binding” maybe “had TFBS most likely bound by NFIX”??

We worded to be explicit it is NFIX motifs that are measured to be differentially bound.

Line 336.
“Cluster 6 was not enriched”

We have made this change.

Line 355
“To investigate the role DNA methylation had on the gene expression changes” rephrase, ask first IF it has a role.

We have changed this to “To investigate if DNA methylation played a role in the gene expression changes”.

Line 356.
What is the average coverage in the genome?

We added details on the consensus set of CpGs which included minimal read coverage of 10x.

Line 355.
Several sentences start with numbers in this paragraph. Rephrase.

We have revised these sentences.

Line 378
Rephrase “An important feature of smoltification is how the process prepares” maybe “An important question about S is how…”

This first statement is describing the preparatory nature of smoltification, not asking the question of how it works. This has been revised to be phrased less as a question and more as a statement.

Line 381
What kind of substance is “NKA a1b”? Does this show up in the liver?

This is the Na+/K+ ATPase α1b isoform which pumps ions out of the gills. This does not play the same role in the liver.

Line 383.
What happens to lipid metabolism. Goes up or down?

We are unsure what this comment refers to. In the same sentence it is stated “lipid metabolism related gene expression is known to decrease in liver of seawater stage Atlantic salmon”.

Line 415
Check statement of First “Yet, no genome wide studies of DNA…” can we reworded. “to our knowledge published”??

We have changed this to “Yet, to our knowledge no genome wide studies of DNA methylation, chromatin accessibility, and TFs involved in driving these transcriptional and physiological changes have been published”.

Line 436
Check wording, something off “with a decrease in peak accessibility of regions near many of these genes”

Changed “was associated” to “coincided”.

Line 474
Add liver in the sentence somewhere.

Added “in liver of Atlantic salmon”.

Line 481
Rephrase “history likely does not play a major role” maybe “history is unlikely to play a major role”

We have made this change.

Line 482
Reword “contrast from studies in gill.”

Changed to “unlike”.

Line 503.
Reword. “Sampling began 21 weeks after first 503 feeding, here called week 1”

We have made this change.

Line 523
“of at least eight was used to make RNA-seq libraries” How many samples failed this test?

No samples had a RIN less than 8. This is the minimum quality of RNA used in this study.

Line 530
“read alignments “ to “alignment of reads”

We have made this change.

Line 533.
Provide hyperlink nf-co.re/rnaseq.

We have added a hyperlink.

Line 539
Strange wording, fix “Differentially expressed genes (DEGs) were tested for first differences across all experimental groups?”

Removed “first” and changed to “short photoperiod group time points”.

Line 542
Strange wording, fix “DEGs were chosen from the results using an FDR cutoff of”

Changed to “DEGs were found using an FDR cutoff of”.

Line 542
“Euclidean distances” calculated with what?

We have added the R function used.

Line 546
Check wording ”DEGs were also tested between experimental and control groups”

Change this to “Differential expression analysis was also performed between”.

Line 553
Was this done directly when sample taken, or retrieved from RNA later? “Two replicate liver tissue samples were used …”

The liver samples were immediately processed after sampled for each timepoint. The text has been revised to make this clear.

Line 567
Reference for “nf-core atacseq pipeline (v1.2.1),”?

Added the DOI to the version. Did the same for the RNAseq pipeline.

Line 571
Wording “computed through the pipeline” is a bit peculiar. Can you rephrase. Phrase used elsewhere also.

Changed “computed through” to “obtained from”.

Line 565
Check wording. “combined with the consensus annotated peak file from the pipeline, annotating peaks to genes by the shortest distance to gene TSS.” In general, check wording of all newly added segments. They tend to be the most troublesome. Like the following sentence “We further associated with the differential accessible peaks the set of DEGs determined”

This has been reworded to improve clarity.

Line 581
Reword “TF footing in the unified ATAC peak set previously generated was done using”

We have slightly reworded this sentence.

Line 584
During the “protocol”?? “locally blocking transposase activity during the ATAC protocol.”

This sentence has been restructured to be more clear.

Line 586
Reword “o associate these footprints with specific TFs.”

Changed to “to associate identify TFs associated with these footprints.”

Line 588
Perfect for supplemental data that other researchers could use. “(generated in-house).”

We used RepeatMasker to mask simple repetitive regions in the genome from the analysis. We have updated the methods section to reflect this.

Line 596.
How was the overlap tested “We tested…”? Which statistical environment, how automated over the whole dataset?

We used a Fisher’s exact test. This is already mentioned in the paragraph (line 608) so we have not modified the text.

Line 608
“Livers from four fish per time point (three fish for week 25) were” Are these samples from the same fish as the RNA and ATAC seq where done on? Indicate in results and in beginning of these sections if these are the same or not.

These were not the same fish, but they were sampled at the same time as the fish used for RNAseq and ATACseq. We have added this detail to the methods and results sections.
